# Ionizing Irradiation Not Only Inactivates Clonogenic Potential in Primary Normal Human Diploid Lens Epithelial Cells but Also Stimulates Cell Proliferation in a Subset of This Population

**DOI:** 10.1371/journal.pone.0098154

**Published:** 2014-05-19

**Authors:** Yuki Fujimichi, Nobuyuki Hamada

**Affiliations:** Radiation Safety Research Center, Nuclear Technology Research Laboratory, Central Research Institute of Electric Power Industry (CRIEPI), Komae, Tokyo, Japan; University of Delaware, United States of America

## Abstract

Over the past century, ionizing radiation has been known to induce cataracts in the crystalline lens of the eye, but its mechanistic underpinnings remain incompletely understood. This study is the first to report the clonogenic survival of irradiated primary normal human lens epithelial cells and stimulation of its proliferation. Here we used two primary normal human cell strains: HLEC1 lens epithelial cells and WI-38 lung fibroblasts. Both strains were diploid, and a replicative lifespan was shorter in HLEC1 cells. The colony formation assay demonstrated that the clonogenic survival of both strains decreases similarly with increasing doses of X-rays. A difference in the survival between two strains was actually insignificant, although HLEC1 cells had the lower plating efficiency. This indicates that the same dose inactivates the same fraction of clonogenic cells in both strains. Intriguingly, irradiation enlarged the size of clonogenic colonies arising from HLEC1 cells in marked contrast to those from WI-38 cells. Such enhanced proliferation of clonogenic HLEC1 cells was significant at ≥2 Gy, and manifested as increments of ≤2.6 population doublings besides sham-irradiated controls. These results suggest that irradiation of HLEC1 cells not only inactivates clonogenic potential but also stimulates proliferation of surviving uniactivated clonogenic cells. Given that the lens is a closed system, the stimulated proliferation of lens epithelial cells may not be a homeostatic mechanism to compensate for their cell loss, but rather should be regarded as abnormal. This is because these findings are consistent with the early *in vivo* evidence documenting that irradiation induces excessive proliferation of rabbit lens epithelial cells and that suppression of lens epithelial cell divisions inhibits radiation cataractogenesis in frogs and rats. Thus, our *in vitro* model will be useful to evaluate the excessive proliferation of primary normal human lens epithelial cells that may underlie radiation cataractogenesis, warranting further investigations.

## Introduction

The ocular lens is a transparent, avascular tissue that refracts incoming light onto the retina and grows throughout life without developing tumors [1]. The lens capsule, lens epithelium, lens cortex and lens nucleus compose the lens, and the boundary between its anterior and posterior surfaces is called an equator. The lens epithelium comprises a single layer of cuboidal epithelial cells located in the anterior subcapsular region. Lens epithelial cells in the germinative zone around the equator divide, migrate posteriorly, and terminally differentiate into fiber cells that possess no organelles [2]. Newly formed fibers wrap around existing cortical fibers, and become more internalized and tightly packed mature nuclear fibers. The lens capsule encases the entire lens, so that all cells stay inside the lens throughout life.

A cataract is a clouding of the lens. Posterior subcapsular (PSC) cataracts are one of the three major types of cataracts, and most common in ionizing radiation-induced cataracts. Such radiogenic cataracts have been described for over a century [3] and regarded as typical late effects of radiation. The International Commission on Radiological Protection (ICRP) considers that the lens is among the most radiosensitive tissues [4]. ICRP has recommended dose limits for the lens to prevent vision-impairing cataracts since 1954 [5], because cataracts limit occupational performance and interfere with daily life activities even if surgically curable and not life threatening. In 2011, ICRP recommended reducing occupational dose limit for the lens by a factor of 7.5 [6], which was revised 21 years after the previous revision [7]. Such lowering may affect some medical or nuclear workers (and perhaps even some patients as well), thereby creating a surge of interest in cataracts [8]. From a therapeutic viewpoint, 10 Gy and 18 Gy are considered as tolerance dose that causes cataracts requiring surgical intervention in 5% and 50% of patients within 5 years post therapy, respectively [9] (c.f., ICRP considers 0.5 Gy as a threshold dose that causes vision-impairing cataracts in 1% of exposed individuals with >20 years follow-up [6]), and treatment planning is made to minimize the lens dose. Nonetheless, children with retinoblastoma are often treated with radiation due to its radiosensitive nature, and this leads to cataracts for which pediatric surgery is a challenge [10]. Manned space missions also raise a concern for cataracts [11]. Despite such a long history documenting radiogenic cataracts, the underlying mechanisms remain unclear, and mitigators are yet to be established [6].

A colony formation assay has been the most extensively used technique in the field of radiation biology since its establishment in 1956 [12]. This assay evaluates the radiosensitivity in terms of the clonogenic survival, where a cell that retains the ability to form a colony containing ≥50 cells (referred hereinafter to as a clonogenic colony) typically in two weeks (a period that varies with cell type) is regarded as a surviving cell. In these experiments, fibroblasts have been most widely used among various types of primary normal human diploid cells. Conversely, there has been no information available hitherto as to the clonogenic survival of lens epithelial cells (i.e., the only possible clonogenic population within the lens structures), which was evaluated here in parallel with fibroblasts for comparison.

## Materials and Methods

### Cell Cultures

The ScienCell Research Laboratories (Carlsbad, CA) sells several lots of primary normal human lens epithelial cells derived from different donors under the same name (HLEpiC) and catalog number (6550). Of these, the lot 5971 (cytokeratin 18-positive HLEpiC harvested from a 24-week gestation female fetus and subsequently passaged once during which time 5 population doublings occurred *in vitro* before shipping) was purchased, and renamed HLEC1 to avoid possible confusion that our future use of different lots of HLEpiC may cause. All flasks and dishes used for HLEC1 cells were precoated with poly-*L*-lysine (PLL), and HLEC1 cells were routinely subcultured in EpiCM [epithelial cell culture medium containing 2% fetal bovine serum (FBS), 1% growth supplement (components not disclosed by the manufacturer), 19 U/ml penicillin and 19 µg/ml streptomycin, pH of 7.4 with an atmosphere of 95% air/5% CO_2_] in 75-cm^2^ tissue culture (T75) flasks, following the manufacturer’s instruction. HLEC1, PLL and EpiCM were purchased from ScienCell. WI-38 primary normal human diploid lung fibroblasts were established by Hayflick as his 38th fibroblast strain at the Wistar Institute of Anatomy and Biology (Philadelphia, PA) in 1962, and derived from a surgically aborted three-month gestation Caucasian female fetus [13], [14]. WI-38 cells that underwent 19 population doublings *in vitro* were purchased from American Type Culture Collection (CCL-75, Manassas, VA), and passaged in T75 flasks in Dulbecco’s modified Eagle’s medium (DMEM, D8437, Sigma, St Louis, MO) supplemented with 10% heat-inactivated FBS (171012, Nichirei Bioscience, Tokyo, Japan). All cell cultures were maintained at 37°C in a humidified atmosphere of 5% CO_2_ in air, except where otherwise specified.

Primary normal human cells have a finite replicative lifespan *in vitro* as firstly proposed by Hayflick and Moorhead in 1961 [15]. To evaluate this, cells were serially passaged. The population doubling number (PDN) was calculated as log_2_(*N*_H_/*N*_P_), where *N*_P_ and *N*_H_ are cell numbers plated and those harvested, respectively. The cumulative population doubling (CPD) level was calculated as the initial PDN (i.e., 5 for HLEC1 and 19 for WI-38) plus the PDN increased by additional passages. The end of the replicative lifespan was defined by failure of the population to increase after a minimum of three weeks in culture with weekly refeedings. The population doubling time was calculated as the reciprocal of *q*, when growth curves were fitted against the data points in the exponential growth phase to *y* = *qx*+*s* where *y*, *x*, *q* and *s* are CPD, time (h), slope and intercept, respectively.

### Preparation of Metaphase Chromosome Spreads

Exponentially growing cells were treated with 25 ng/ml colcemid (Gibco, Grand Island, NY). After mild trypsinization and mitotic shake off, cells were hypotonized with 75 mM KCl, fixed in 3∶1 methanol:acetic acid, dropped onto cleaned slides, and stained with 6% Giemsa (Wako, Osaka, Japan). Chromosome numbers in each metaphase were counted under an Olympus BX51 microscope.

### Irradiation and Colony Formation

At one day prior to irradiation, 1.8×10^5^ cells were seeded onto each T25 flask. Cells were exposed to single, graded doses of X-rays at room temperature from an X-ray generator (MBR-1505R2, Hitachi Medico, Tokyo, Japan) operated at 150 kV and 5 mA with a 1-mm aluminum plus 0.2-mm copper filter, followed by incubation at 37°C in 95% air/5% CO_2_. Only one T25 flask was placed at a time in a single exposure in the central 15.8-cm-diameter circle at the distance from the radiation source to the cell surface of 250 mm, and irradiated at a dose rate of 0.43±0.005 Gy/min for HLEC1 and 0.43±0.008 Gy/min for WI-38. The dose uniformity within the T25 flask was ±4.9%, and the half value layer of X-rays used was 5.5 mm. Control cells were sham-irradiated and manipulated in parallel with the test cells. Within 1 h postirradiation, cells were rinsed, trypsinized, and suspended in EpiCM (for HLEC1) or DMEM with 10% FBS (for WI-38). Then, cells were counted, diluted in DMEM with 20% FBS, and reseeded into 10-cm dishes in quadruplicate, and incubated for 14 days, at which time they were fixed and stained with crystal violet. Stained cells were viewed under a Nikon SM2645 stereomicroscope, and the 8-bit RGB color JPEG images were captured with a Panasonic DMC-TZ30 digital camera. Note that the difference in cell numbers replated after irradiation for colony formation was statistically insignificant between HLEC1 and WI-38 (Table S1).

Hereafter, a colony with 2–49 cells is referred to as an abortive colony, a cell capable of producing a clonogenic colony as a clonogenic cell, and a cell incapable of producing a clonogenic colony as a nonclonogenic cell. The plating efficiency of sham-irradiated cells was calculated as the number of clonogenic colonies divided by that of plated cells. The surviving fraction of irradiated cells was calculated as the number of clonogenic colonies divided by that of plated cells with correction for the plating efficiency. Survival curves were fitted against the means of independent experiments to the exponential equation *y* = exp (−*kx*), where *y*, *x* and *k* are surviving fraction, the dose and slope, respectively. The dose required to reduce the surviving fraction to 0.1 (10% survival dose, *D*_10_) and that to 1/*e* (mean lethal dose, *D*_0_) was calculated as [ln(1/0.1)]/*k* and 1/*k*, respectively.

To quantify differences among clonogenic colonies, colony size was evaluated based on the methods explained in Figures S1, S4, S5 and S6. Briefly, cell numbers in all manually countable clonogenic colonies were directly counted under the stereomicroscope, and this was the case for most colonies with <1,500 cells. The area of each clonogenic colony, and cell numbers in manually uncountable clonogenic colonies were evaluated using the captured images and the ImageJ 1.47 freeware (http://rsb.info.nih.gov/ij/).

### Statistical Analysis

Data were calculated as the means and standard deviations (SD) of three repeated experiments unless otherwise described. Statistical comparisons between groups were made by Student’s *t*-test, and a *p* value of <0.05 was considered to be significant.

## Results

### HLEC1 and WI-38 are Diploid, and HLEC1 has a Shorter Replicative Lifespan than WI-38

**Figure 1:**
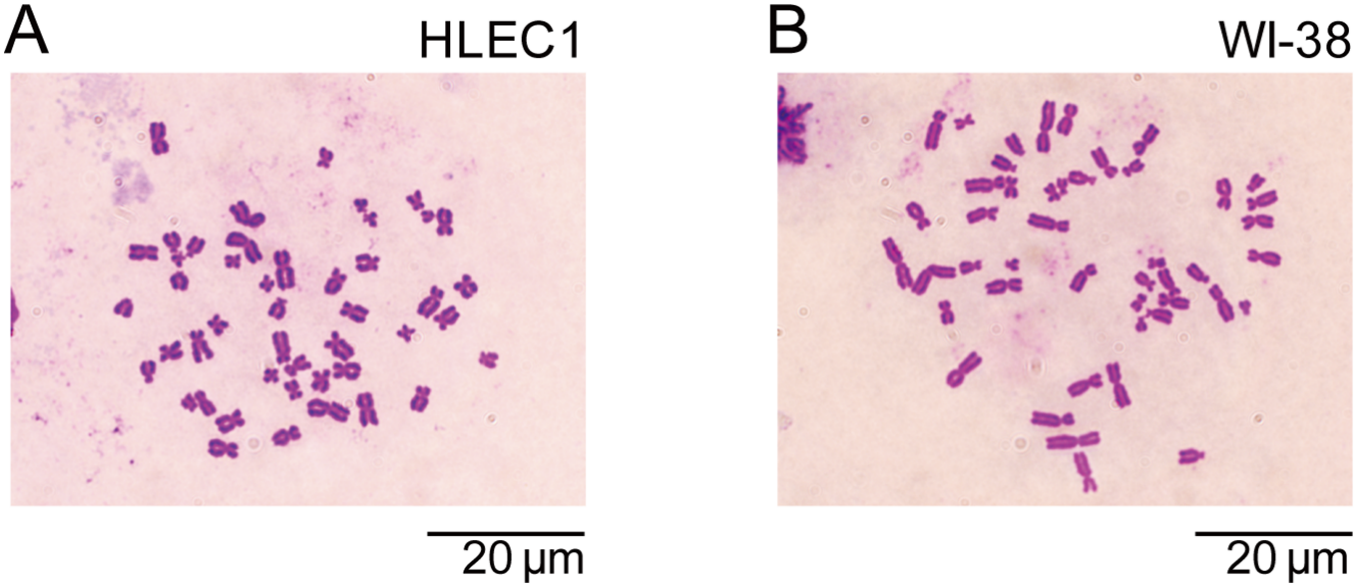
Representative images of metaphase chromosomes in HLEC1 at CPD 14.5 (A) and WI-38 at CPD 44.7 (B). Bars, 20 µm.

**Figure 2:**
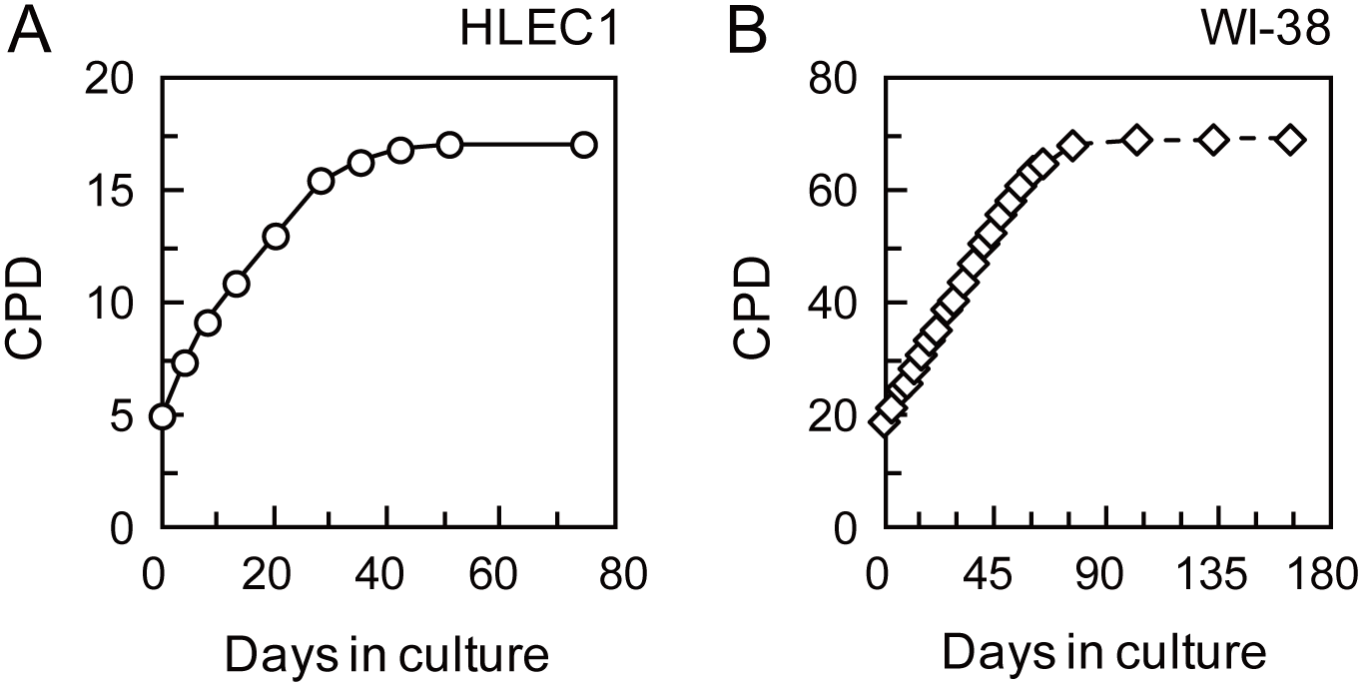
Growth curve of HLEC1 (A) and WI-38 (B). HLEC1 at CPD 5.0 and WI-38 at CPD 19.0 were plated and serially passaged with weekly replenishment. The population doubling times of HLEC1 and WI-38 were estimated to be 66.6 h at CPD 5.0–15.5 and 32.4 h at CPD 19.0–64.9, respectively. HLEC1 and WI-38 ceased to divide at CPD 17.1 and 69.1, respectively.

Cells need to go through a minimum of 5.64 doublings for clonogenic colony formation, but the proliferative potential of HLEC1 lens epithelial cells is uncharacterized nor is its ploidy unlike the case for WI-38 lung fibroblasts [13]. To this end, we evaluated the ploidy and proliferative potential. The karyotype analysis of metaphase chromosome spreads confirmed that both strains are diploid (Table 1 and Figure 1). Serial passages revealed that HLEC1 and WI-38 have a population doubling time of 66.6 and 32.4 h, and reach the end of their replicative lifespan at CPD 17.1 and 69.1, respectively (Figure 2). Young cells (i.e., CPD 11.0 for HLEC1 and CPD 32.0 for WI-38) were thus used for subsequent experiments.

**Table 1:** Karyotype.

	Cell types	
	HLEC1	WI-38
CPD at the time of analysis	14.5	44.7
Number of metaphases analyzed	52	66
Number of chromomosomes/cell	46	46

### Clonogenic Survival of HLEC1 and WI-38 Similarly Declines with Radiation Dose, Whilst HLEC1 has a Lower Plating Efficiency than WI-38

**Figure 3:**
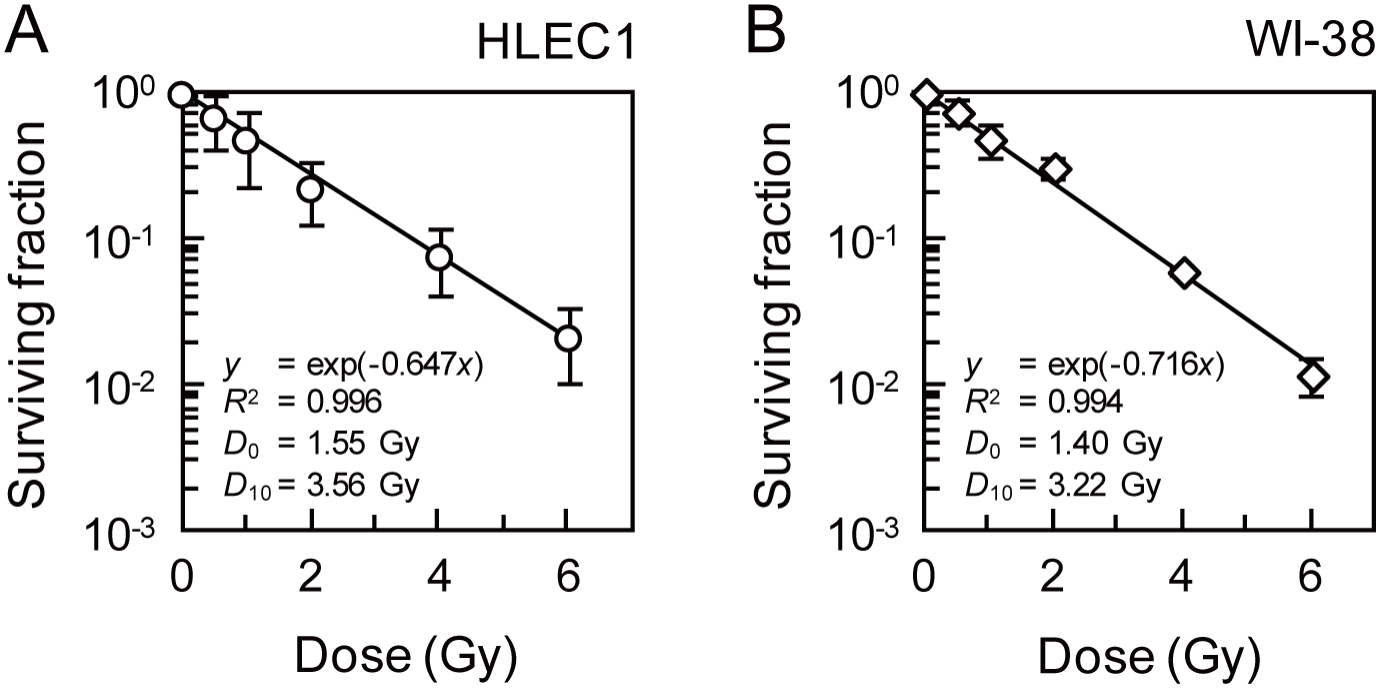
Clonogenic survival of HLEC1 (A) and WI-38 (B). Within 1 h after exposure to indicated dose of X-rays at a dose rate of 0.43±0.01 Gy/min, cells were reinoculated for colony formation and incubated for 14 days at which time they were fixed and stained. At the time of reinoculation, CPD of HLEC1 and WI-38 was 11.0 and 32.0, respectively (n.b., CPD 11.0 was experimentally available youngest HLEC1 in our hand). The plating efficiency of HLEC1 and WI-38 was 1.6±0.6 and 19.5±3.6%, respectively. Curves were fitted to the exponential equation *y* = exp (−*kx*), and *D*_0_ and *D*_10_ were calculated as 1/*k* and [ln(1/0.1)]/*k*, respectively. *R* ^2^, correlation coefficient square. The data are presented as means and SD of three independent experiments with quadruplicate measurements.

HLEC1 at CPD 11.0 and WI-38 at CPD 32.0 were reinoculated within 1 h postirradiation, followed by colony formation for 14 days. Note that CPD 11.0 was the youngest HLEC1 experimentally available to us. The colony formation assay revealed that the clonogenic survival similarly decreases with increasing dose of X-rays in both strains (Figure 3). Whereas the plating efficiency of HLEC1 was significantly lower than that of WI-38 (1.6±0.6% vs 19.5±3.6%, *p* = 0.0056), the difference between these two survival curves was statistically insignificant at all five dose points tested (i.e., 0.5–6 Gy) for which *p* values ranged from 0.11 to 0.50. These results suggest that whilst HLEC1 has a smaller fraction (∼one-twelfth) of clonogenic cells than WI-38, irradiation with the same dose inactivates the same fraction of clonogenic cells in both strains.

### Irradiation Stimulates the Proliferation of Surviving Uninactivated Cells in HLEC1 but not in WI-38

**Figure 4:**
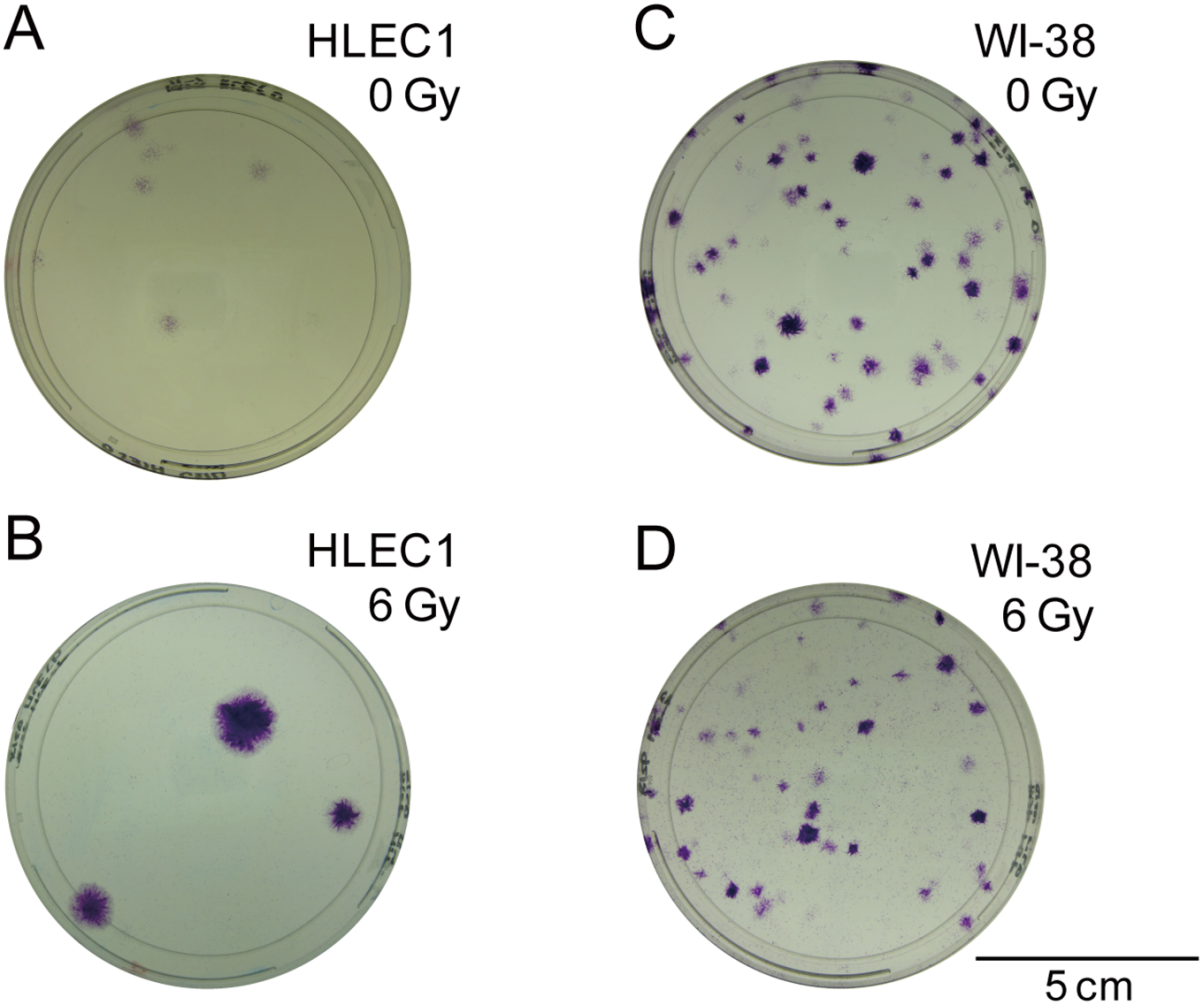
Representative images of colonies arising from sham-irradiated HLEC1 (A), 6 Gy-irradiated HLEC1 (B), sham-irradiated WI-38 (C), and 6 Gy-irradiated WI-38 (D). Shown are colonies that were formed during 14 days in 10-cm dishes and stained with crystal violet. Bar, 5 cm.

Interestingly, the colony formation assay also led us to the observation that clonogenic colonies arising from irradiated HLEC1 look obviously larger than those from sham-irradiated counterparts (Figure 4). This potentially challenges the long-held tenet among users of the colony formation assay that irradiation suppresses proliferation and increases killing of cells thereby resulting in dose-dependent clonogenic inactivation [3], [12]. We hence decided to quantify these differences in clonogenic colonies.

**Figure 5:**
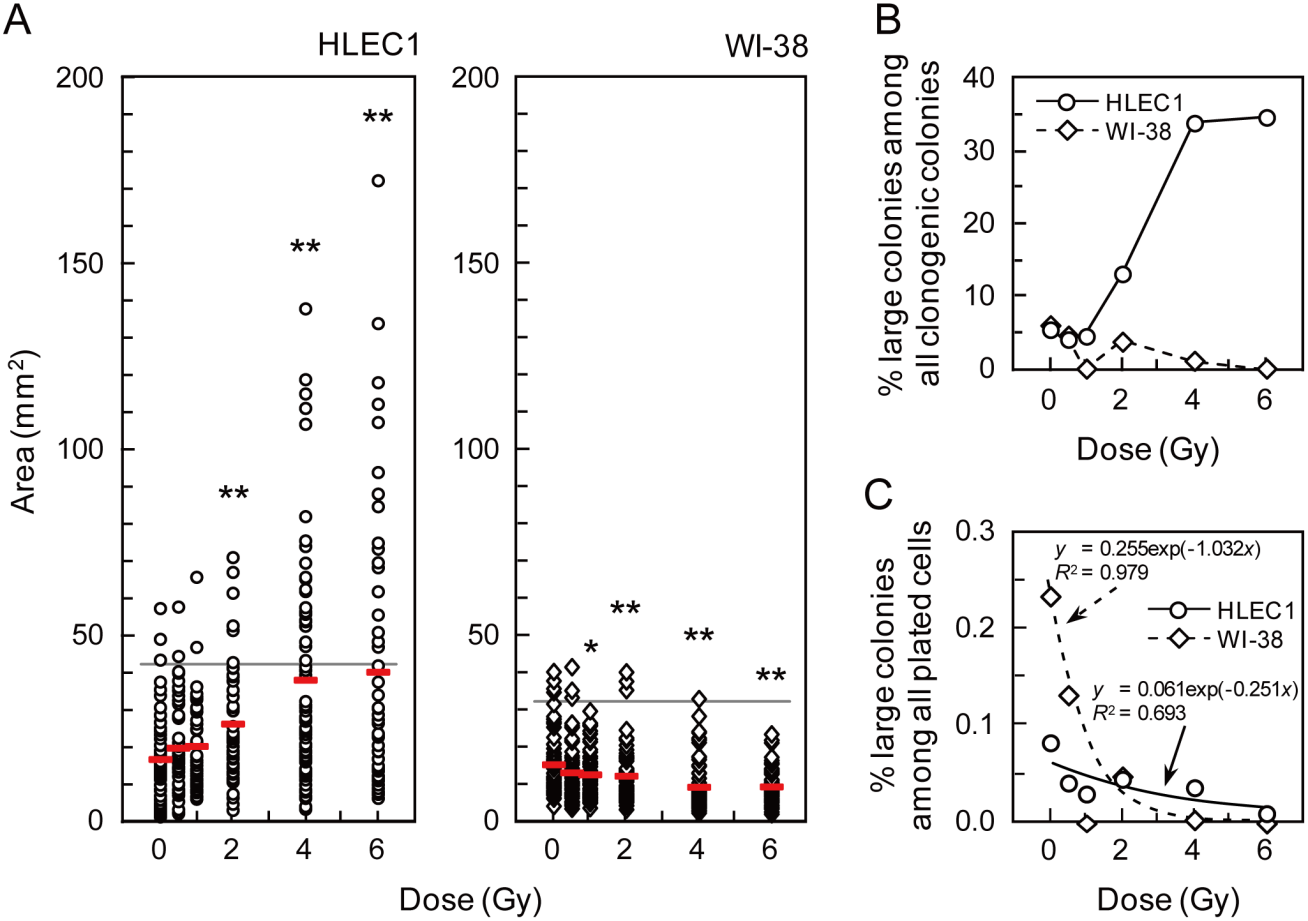
The area of clonogenic colonies. (A) The area distribution of clonogenic colonies arising from HLEC1 (left panel) and WI-38 (right panel) as a function of dose. The area of clonogenic colonies (45–68 colonies for HLEC1 and 54–93 colonies for WI-38 per dose point) was measured as described in Figure S1. Red bars indicate the means at each dose point. The horizontal lines indicate the mean+2SD area in sham-irradiated controls (41.7 mm^2^ for HLEC1 and 31.7 mm^2^ for WI-38). *0.01≤*p*<0.05 and **p<0.01 compared with sham-irradiated controls. See Figure S2 for the frequency distribution replotted as a function of the area. (B) The fraction of clonogenic colonies exceeding the mean+2SD area of sham-irradiated controls among all clonogenic colonies analyzed. (C) The fraction of clonogenic colonies exceeding the mean+2SD area of sham-irradiated controls among all plated cells. Curves were fitted to the exponential equation, though the data points for 1 Gy and 6 Gy for WI-38 were excluded because of zero values. *R* ^2^, correlation coefficient square. When the intercept of the exponential curve for WI-38 was changed from 0.255 to 1, its slope changed from 1.032 to 0.716, and changed from 0.979 to 0.994. For Figure 5B and 5C, circles with solid lines and diamonds with dotted lines indicate the data for HLEC1 and WI-38, respectively.

To confirm our above-described impression, the area of clonogenic colonies was first analyzed as explained in Figure S1. Figure 5A illustrates that whereas the area of clonogenic colonies derived from HLEC1 and that from WI-38 does not differ significantly at 0 Gy (*p* = 0.24), the former was significantly larger than the latter at ≥0.5 Gy (*p*<0.001). Frequency of clonogenic colonies did not exhibit multimodal distributions for both strains and at all dose points (Figure S2), so that clonogenic colonies exceeding the mean plus 2SD area of sham-irradiated controls were judged as “large” colonies. As regards WI-38, the area of clonogenic colonies decreased significantly at ≥1 Gy compared with sham-irradiated controls (Figure 5A). Decreases were also observed in the fraction of large colonies among all clonogenic colonies and that among all plated cells (Figure 5B and 5C). The exponential equation fitted the latter fraction data well with a correlation coefficient square (*R* ^2^) of 0.979 (Figure 5C). Given a dose-dependent decrease in the size of clonogenic colonies, this is not surprising because the curve of this sort should reflect the clonogenic survival when colonies above the mean plus 2SD area (e.g., with several thousand cells) were judged as survivors instead of a general criterion of 50 cells. These observations obtained with WI-38 reconfirmed the rationale of the colony formation assay, such that irradiation inactivates clonogenic potential and decreases the clonogenic survival both of which occur dose dependently. With respect to HLEC1, the area of clonogenic colonies was elevated with dose, and such increases were significant at ≥2 Gy compared with sham-irradiated controls (Figure 5A). Surprisingly, large colonies accounted for as high as 33.8% and 34.6% of all clonogenic colonies at 4 and 6 Gy, respectively (Figure 5B), whereas its fraction was similar to WI-38 at 0 Gy and 0.5 Gy. When the data for the fraction of large colonies among all plated cells were fitted to the exponential equation, the curve looked much flatter than the case for WI-38 (Figure 5C), and such difference should be attributable to the increased proliferation following irradiation. Refitting of these data at 0.5–4 Gy to the linear equation yielded the line with a slope of 2.6×10^−5^, nearly parallel to the x-axis (Figure S3). These findings suggest that irradiation not only inactivates clonogenic potential but also stimulates cellular proliferation, and imply that such cells whose proliferation is activated by irradiation exist in the population at a similar rate independent of dose.

**Figure 6:**
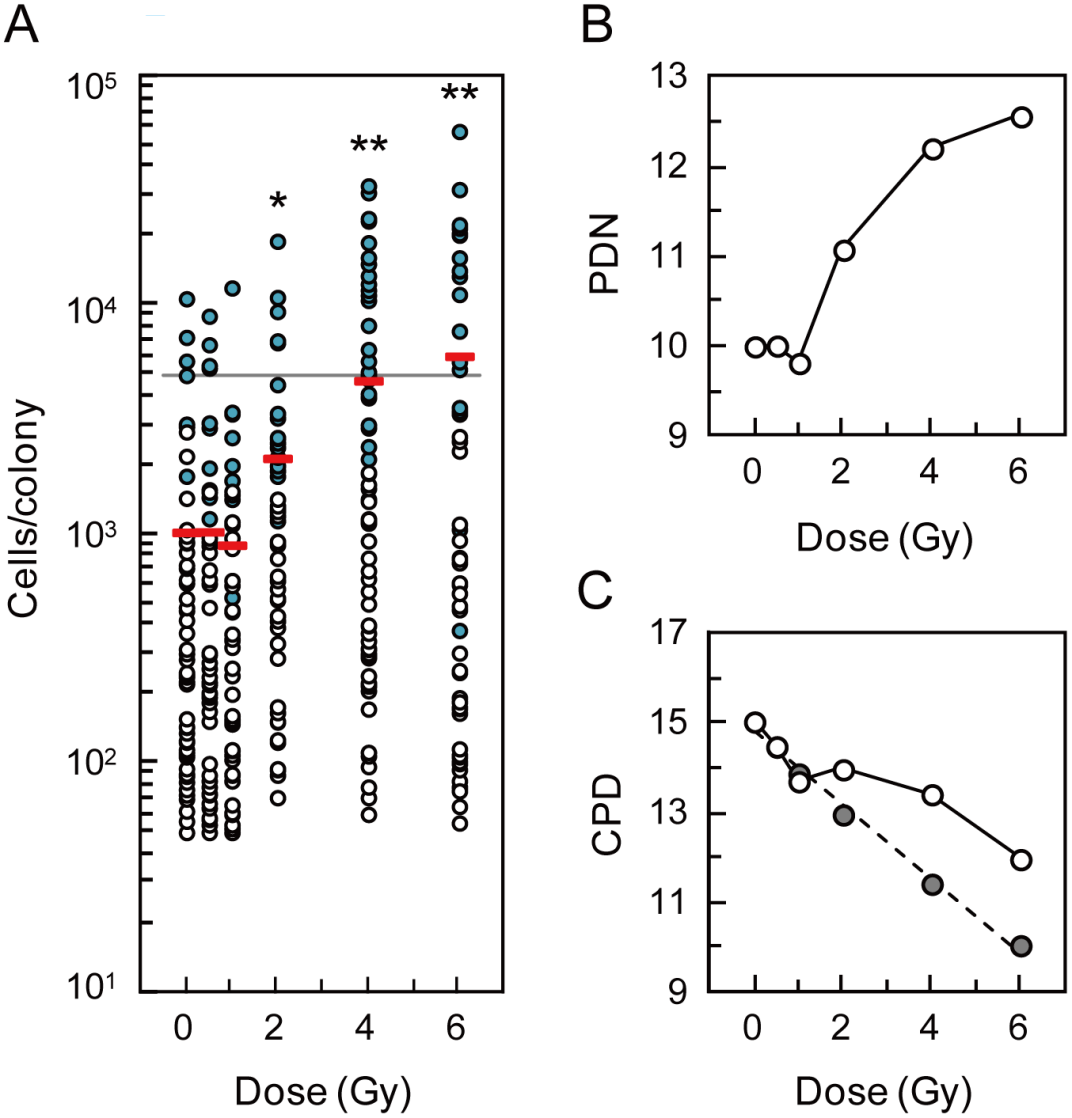
Changes in population doublings of HLEC1. (A) Cell numbers in individual clonogenic colonies. Cell numbers in 218 countable clonogenic colonies (open circles) were directly counted under the stereomicroscope, and those in 99 uncountable colonies (blue-filled circles) were estimated as described in Figures S4, S5 and S6. Red bars indicate the means at each dose point. A horizontal line indicates the mean+2SD value (4,944 cells) in sham-irradiated controls. *0.01≤*p*<0.05 and **p<0.01 compared with sham-irradiated controls. (B) PDN of clonogenic cells experienced during colony formation. To calculate PDN, cell numbers in individual clonogenic colonies (shown in Figure 6A) were summed, divided by the number of clonogenic colonies (i.e., the number of clonogenic cells plated), and then transformed to the logarithm to base 2. (C) CPD of all cells in the whole population. For calculation of the data, cell numbers in clonogenic colonies (shown in Figure 6A) were first summed. Second, the number of all cells situated outside clonogenic colonies (i.e., cells in abortive colonies or other nonclonogenic cells) was directly counted under the stereomicroscope and summed. Third, the sum of these cell numbers was divided by the number of plated cells, and transformed to the base-2 logarithm. Finally, 11.0 (CPD when plated) was added to these numbers, and shown as open circles with a solid line. Gray-filled circles with a dotted line represent CPD of all cells calculated assuming that irradiation does not alter the proliferation of clonogenic cells (i.e., PDN of irradiated clonogenic cells is the same as PDN of sham-irradiated controls regardless of dose).

**Figure 7:**
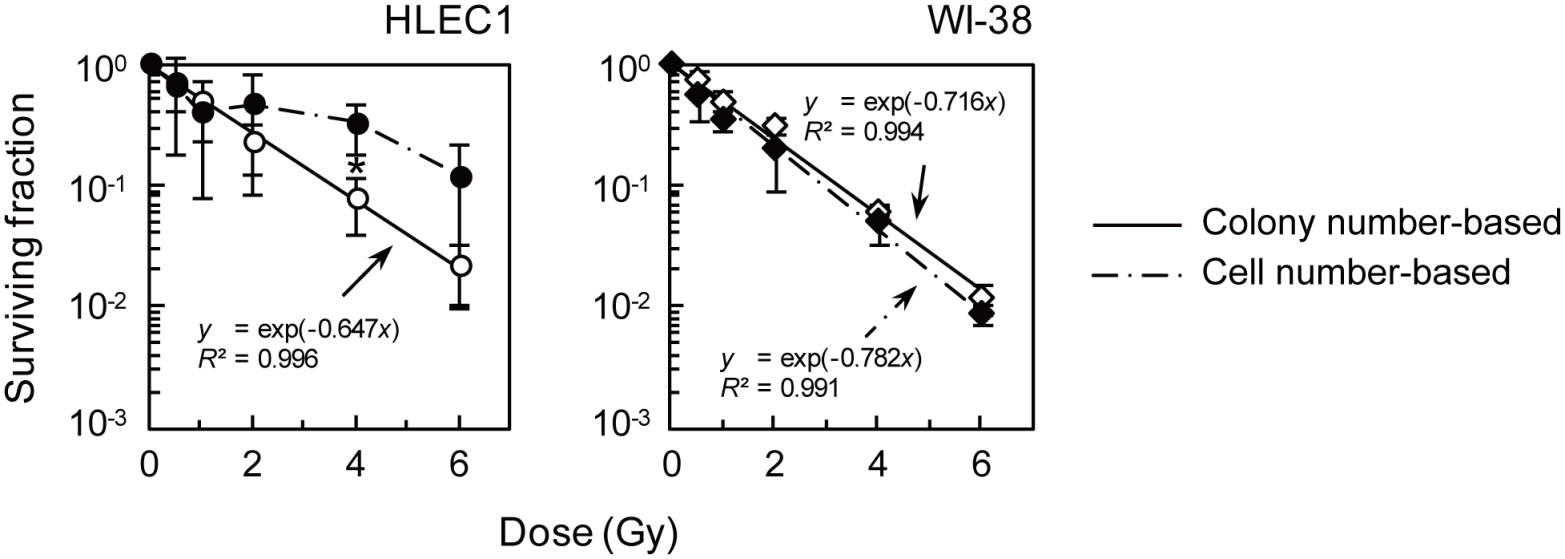
The impact of cell numbers in clonogenic colonies on the surviving fraction in HLEC1 (left panel) and WI-38 (right panel). The general “colony number-based” surviving fraction was calculated from the number of clonogenic colonies divided by the number of plated cells with correction for the plated efficiency. This was compared here with the “cell number-based” surviving fraction that was calculated as the sum of the integrated density of clonogenic colonies (i.e., cell numbers in clonogenic colonies given a linear relationship between the integrated density and cell numbers in each clonogenic colony) divided by the number of plated cells with correction for that at 0 Gy. Colony number-based survival curves (open symbols with solid lines) were taken from Figure 3, and the data of the integrated density of clonogenic colonies presented in Figure S5 were used to obtain cell number-based curves (closed symbols with dotted lines). The data represent means and SD of three independent experiments with quadruplicate measurements. *0.01≤*p*<0.05 compared between two types of survival curves at each dose. For WI-38, the difference between the colony number-based survival curve and the cell number-based survival curve was insignificant for all 5 dose points tested (*p*>0.10), indicating that the colony number-based survival fraction is akin to the cell number-based one. For HLEC1, the difference between two types of survival curves reached a statistical significance only at 4 Gy (*p* = 0.02), but there was clearly a tendency toward the increased surviving fraction at 2 Gy and 6 Gy.

Cell numbers in clonogenic colonies arising from HLEC1 were further evaluated as described in Figures S4, S5 and S6, because the increased colonial area may not necessarily reflect the increased proliferation. Cell numbers in clonogenic colonies significantly increased at ≥2 Gy, and ranged from 50 cells/colony (i.e., criteria for a survivor in the colony formation assay) observed at 0, 0.5 and 1 Gy to 57,745 cells/colony observed at 6 Gy each of which corresponded to PDN 5.64 and 15.8, respectively (Figure 6A). Figure 6B clearly shows that irradiation greatly increases PDN of clonogenic cells during a period of 14 days for colony formation (e.g., PDN 12.6 at 6 Gy increased from PDN 10.0 at 0 Gy). This finding reinforces the observation that irradiation stimulates the proliferation of clonogenic cells, but the impact of irradiation on nonclonogenic cells was still unclear. To address this, the number of all cells outside clonogenic colonies (i.e., cells in abortive colonies or other nonclonogenic cells) was counted. Then, the changes in CPD of the whole population were reasonably evaluated (Figure 6C), such that CPD in sham-irradiated controls that actually increased after 14 days (>4) was not so different from that assumed given a population doubling of 66.6 h (<5). As a whole population, a replicative lifespan (∼17.1 CPD) was not exceeded irrespective of dose, and irradiation reduced population doublings. The CPD for HLEC1 was less reduced at high dose due to cellular proliferation in clonogenic colonies than WI-38 cells (Figure 6C), and this led to changes in the shape of the survival curve for HLEC1 (Figure 7).

## Discussion

Here we have carried out the colony formation assay of two primary normal human diploid female fetal cell strains: HLEC1 lens epithelial cells and WI-38 lung fibroblasts. HLEC1 had the lower plating efficiency than WI-38, but a difference in the clonogenic survival (colony number-based survival) between two strains was insignificant (Figure 3), indicating that the same dose inactivates the same fraction of clonogenic cells in both strains. For WI-38, the fraction and size of clonogenic colonies declined with dose to a parental cell (Figures 5A and S5), and a difference between the colony number-based survival and the cell number-based survival was insignificant (Figure 7). These findings conform to the general principle of the colony formation assay [12] and are also consistent with our previous findings obtained with AG01522D primary normal human diploid male nonfatal foreskin fibroblasts [16], [17], [18]. Intriguingly, however, the reverse held true for HLEC1. The area of clonogenic colonies and its cell numbers escalated significantly at ≥2 Gy (Figures 5A and 6A), and the “large” colonies explained nearly one-third of all clonogenic colonies at 4 and 6 Gy (Figure 5B). It is evident from these findings that irradiation stimulates the proliferation of surviving uninactivated clonogenic cells. Findings obtained with HLEC1 are discussed in more detail below.

### The Results of Cell Numbers and PDN may be Underestimated

The colony formation assay is unable to exactly determine cell numbers, the temporal kinetics of cell divisions, and the doubling number of individual cells. This is not only because some cells are lost while cells are washed, fixed and stained after colony formation, but also because the assay does not provide the information on the timing of each event (e.g., death and proliferation) that occurs during colony formation. Cells to be lost should consist of spontaneously occurring “nonattached” cells, and radiation-induced detached/floating cells due to death, inactivation and/or mitosis of irradiated cells and their progeny cells. Of these, spontaneous cell loss should partly be reflected in the plating efficiency, but radiation-induced cell loss should rise with dose. Pertinently, PDN was calculated by transformation of the relative cell numbers (e.g., cell numbers at days 0 versus 14) to the base-2 logarithm. However, the kinetics of cells with *n* divisions should not always obey the *n*-th power of 2, and should be more complicated such as by delayed reproductive death that occurs dose dependently and explained by a branching process model [16], [17], [18], [19]. Accordingly, these should cause underestimation of cell numbers, PDN and individual cell divisions at high dose. In other words, it seems likely that compared with the present results, cells experienced more divisions and colonies contained more cells when parental cells received high dose.

The computerized video time-lapse analysis may overcome limitations of the colony formation assay such as those just discussed above, but still has difficulty in tracing the fate of all cells during the entire colony formation process (e.g., due to limited capacity and occasional cell loss from the field of view [20]). This awaits further development of a time-lapse analysis system, but the colony formation assay would serve as a feasible and easy technique to know the fate of irradiated cells and their descendants.

### Stimulated Proliferation Should be Regarded as Excessive Rather than Compensatory

Irradiation increased PDN of the clonogenic cell population during 14 days (e.g., 2.6 population doublings at 6 Gy additional to 0 Gy, Figure 6B). The most pronounced clone with 15.8 doublings at 6 Gy was estimated to have a doubling time of 21.0 h given a constant growth rate during 14 days (c.f., the population doubling time of 66.6 h presented in Figure 2). As a whole, CPD of the whole cell population (nonclonogenic plus clonogenic cells) during colony formation did not exceed the replicative lifespan of ∼CPD 17.1 (Figures 2 and 6C). The CPD of the irradiated population was less than the unirradiated control, but would have been even more reduced if surviving uninactivated cells had not been stimulated to divide (Figure 6C). In general, such stimulated proliferation may act as a homeostatic, preventive mechanism to compensate for cell loss, but this should not be the case for the lens. This is because the lens is a closed system inside which all live and dead cells stay throughout life. Namely, not only cells in clonogenic colonies but also all of other cells (e.g., cells in abortive colonies, and detached cells) observed in our *in vitro* system would not go outside the lens *in vivo*, though its accurate numerical data cannot be obtained as discussed above. Thus, the stimulated proliferation of the lens epithelial cells should be regarded as the abnormal, excessive proliferation.

Incidentally, the clonogenic survival of HLEC1 and WI-38 did not differ significantly, when a general criterion of 50 cells was used to define survivors (Figure 3). For WI-38, this survival was commensurate with the survival for which a criterion of the mean plus 2SD area was used (Figure 5C) as well as with the cell number-based survival (Figure 7). This is somewhat reminiscent of our previous result with AG01522D that the survival does not much vary when a criterion of 10 to 100 cells was used [18]. For HLEC1, however, these curves looked different (Figures 5C and 7) because of the stimulated proliferation, so that a general criterion of 50 cells should be used.

### What is the Potential Etiological Significance of the Excessive Proliferation?

Lens epithelial cells in the germinative zone of the lens epithelium around the equator divide [2]. Rodent cataracts have been reported to occur after the equatorial region was locally irradiated [21], but not after irradiation when the germinative zone was shielded [22], [23], [24], indicating that cells in the germinative zone are the relevant cells at risk. To our knowledge, only a few papers [25], [26], [27] reported the radiation-induced excessive proliferation of lens epithelial cells, ahead of this work Dating back to the 1950s, von Sallman et al. observed that excessive proliferation of lens epithelial cells in the germinative zone within 21 days after X-irradiation of the rabbit eye occurred slightly at 125 r, became evident at 250 r and more manifested at higher dose (tested up to 2,000 r) [26]. Likewise, Pirie and observed overshoot of mitosis, which was maximal at 3–4 weeks and returned to a control level at 6–8 weeks after irradiation of the rabbit eye with 1400 r or 1600 r of X-rays [27]. Whereas they studied rabbit lens *in vivo*, we used HELC1 human cells *in vitro* where the microenvironment of their *in vitro* growth system for studying the HLEC1 radiation response may not necessarily mimic the normal growth signaling milieu of migrating lens epithelial cells within the capsule *in vivo*. Despite such great difference in experimental approaches, the findings obtained were surprisingly consistent especially in terms of dose range, considering that 1 Gy roughly equals 100 r. Taken together, there is evidence that inhibition of lens epithelial cell divisions prevents the occurrence of radiogenic cataracts in frogs and rats [28], [29], [30], showing that dividing lens epithelial cells are the target of radiation cataracts. All together, these findings suggest that excessive proliferation of lens epithelial cells in the germinative zone underlies radiation cataractogenesis, and highlight that our *in vitro* model system will be useful to evaluate the excessive proliferation of primary normal human lens epithelial cells.

Cell divisions should vary temporally with dose and among clones to produce a clonogenic colony with the same cell number (e.g., one cell grows constantly in 14 days, another cell grows fast only in the first few days, and the other cell grows fast only in the last few days), but the colony formation assay does not provide such information as discussed above. The only available pertinent information comes again from von Sallman et al. [26] who documented that following exposure to 250 or 500 r, mitotic cell counts returned to control level at day 5 and thereafter remain excessive at least up to day 21 [26].

### What is the Nature of Surviving Uninactivated Radioresistant Cells of which Proliferation is Stimulated by Irradiation?

The curve for the fraction of large colonies among all plated cells looked much flatter than the case for WI-38 (Figures 5C and S3), implying that the cells whose proliferation is stimulated by irradiation exist in the population at a similar rate independent of dose. Such surviving uninactivated cells should certainly be radioresistant, but its nature and whether such cells in the irradiated population originate from clonogenic cells or from nonclonogenic cells in the sham-irradiated population are unknown. Nevertheless, if the latter is the case, it is tempting to speculate that a candidate might be slow cycling radioresistant tissue stem or progenitor cells that become rapidly cycling stem cells or give rise to other cell types upon irradiation. There is evidence for such phenomena in highly regenerative tissues like the intestine [31] and for radiation-induced proliferation of intestinal stem cells [32]. In this regard, lens stem cells remain unidentified, but its putative slow cycling stem cells have been reported to locate around the germinative zone of the lens epithelium [33]. Future studies should characterize the nature of lens stem cells and surviving uninactivated radioresistant cells.

Due to the limited number of HLEC1 available for experiments, this study employed the low plating density before irradiation, but this does not duplicate the conditions of the lens epithelium *in vivo* where cells are in close contact. Given that radiation responses depend on cell density in various cell types, it would be important to test if plating density at the time of irradiation affects the phenomena observed here.

Generally, depending on cell cycle phases at the time of irradiation, irradiation with the higher dose causes more prolonged cell cycle arrest and cell death. In contrast, for the radiogenic excessive proliferation, cell cycling may become faster at higher dose (e.g., by shortening G1 and/or G2 phases). Thus, further studies should address how radiation responses of synchronized cells and non-synchronized cells are different, and determine which cell cycle phase is most susceptible for the radiogenic excessive proliferation.

### What are the Implications of the Excessive Proliferation?

Radiation cataract is a typical tissue reaction with a dose threshold below which no effect would occur [6]. This belief has prevailed since 1969 [34], but has been challenged by mounting epidemiological evidence documenting no threshold [6], [8]. This necessitates the elucidation of biological mechanisms. In this respect, it would be important to consider different underlying etiologies, as Worgul et al. mentioned earlier that though the reason for the varying response of epithelial cells to different doses is unknown, it is possible that the production of radiation cataract may involve more than one mechanism [35]. Human radiation cataracts are typical late effects that reportedly take a few months to decades to appear [36], [37]. Common radiogenic cataracts are PSC cataracts, but cortical cataracts have also been associated with radiation exposure [38], [39]. It should be pointed out that whereas a latency period of PSC cataracts greatly varies from months to decades, cortical cataracts only appear many years after exposure. For instance, it was in 2004 that the first description of cortical cataracts in atomic bomb survivors of Hiroshima and Nagasaki emerged [40]. This suggests the different mechanisms for production of early onset cataracts (e.g., PSC cataracts taking months to years to appear) and late onset cataracts (e.g., PSC and cortical cataracts taking years to decades to appear). Early onset cataracts may be accounted for, at least in part, by the excessive proliferation such that whereas complete loss of organelles during lens fiber cell terminal differentiation is responsible for lens transparency [41], excessive proliferation may cause meridional row disorganization (the known essential event associated with cataract severity) and force undifferentiated epithelioid cells to move posteriorly before organelle loss, which may eventuate in PSC opacification [2], [42]. The excessive proliferation of HLEC1 cells observed here was significant at ≥2 Gy (Figures 5A and 6A), and this passably resembles the acute threshold of 0.5–2 Gy for detectable lens opacities and 2–10 Sv for vision-impairing cataracts that ICRP recommended for almost two decades before 2011 [43], [44]. A threshold for early onset cataracts thus sounds biologically plausible. Considering that the lens is a closed system, cells that constitute opacities should not be limited to excessively proliferated cells and its progeny cells, and may include inactivated cells and dead cells. Figure S7 demonstrates that unlike the case for AG01522D fibroblasts where the frequency of abortive colonies rises with dose [16], the frequency of abortive colonies does not much alter at ≤2 Gy and greatly declined (colonies with 8–15 cells disappeared) at 4 and 6 Gy (n.b., due to the different radiosensitivities, the survival at 2 and 6 Gy in HLEC1 roughly corresponds to that at 4 and 8 Gy in AG01522D, respectively). This suggests that irradiated HLEC1 cells except for surviving uninactivated radioresistant cells are relatively “quiet” (dormant, quiescent or slow cycling) because of high sensitivity to radiation-induced inactivation and cell death (e.g., via premature senescence, apoptosis, necrosis and autophagy [45] for which further analysis is under way), and/or because of nonpermanent albeit very long term cell cycle arrest. Such inactivated cells may also move posteriorly (but slower than excessively proliferated cells) contributing to late onset cataract. Descendent cells arising from irradiated cells (e.g., uninactivated cells or very slowly recovered cells, including stem or progenitor cells) may contribute to late onset cataracts. If abnormal cells arising from damaged single cells form cataracts, then no threshold for cataracts may sound biologically plausible. Pertinent to this, a recent report highlights that a lens progenitor-like cell differentiated from human embryonic stem cells generates a lentoid body [46], warranting further extensive studies to test if an irradiated single lens stem cell forms a cloudy lentoid body.

The lens with progressive PSC cataract is smaller in size than that with stationary PSC cataract for the subject’s age (n.b., the analysis included all kinds of etiological agents that induce PSC cataract) [47], further corroborating the key role of cell proliferation in radiation cataractogenesis. One might assume that progressive PSC cataract would have more proliferation than stationary PSC cataract, but the reverse may be true considering the data in Figure 6C where the CPD was reduced even when the excessive proliferation occurred.

There have been no established mitigators for radiogenic cataracts [6], but this situation may be improved if the mechanisms behind the radiation-induced excessive proliferation are revealed. In this light, it should be noted that although primary tumors of the human lens are not known, yet spontaneous cataractogenesis seems to involve a variety of tumor suppressor genes and DNA repair genes (e.g., p53, p16^Ink4a^, p19^Arf^ or p14^Arf^, p27^Kip1^, ATM, RAD9, BRCA1, NBS1, WRN, XRCC1 and HSF4) [1], [8]. These molecules may be candidate contributors as many of them are also key players in cell cycle checkpoints and general radiation responses. Furthermore, evidence is available that p53 prevents spontaneous PSC cataract formation by suppression of cellular proliferation in mice [2]. Taken together, a recent study has documented that p53 delays while p21 drives spontaneous cataractogenesis in BubR1 progeroid mice [48]. Thus, it would be worth testing if enhancement of p53 function suppresses radiation-induced excessive proliferation *in vitro* using our system, followed by *in vivo* testing [2], [8].

## Conclusions

This study is the first to report the clonogenic survival of lens epithelial cells exposed to ionizing radiation, which was evaluated with the colony formation assay. The data clearly showed that irradiation not merely inactivates clonogenic potential dose dependently but even stimulates the proliferation of HLEC1 primary normal human diploid lens epithelial cells. Lines of *in vivo* evidence support the possibility that such excessive proliferation may be one of the mechanisms underpinning radiation cataractogenesis. In contrast to the case for surviving uninactivated radioresistant clonogenic cells that underwent excessive proliferation, irradiated nonclonogenic cells seemed quiet because of high radiosensitivity to inactivation and cell death, and/or because of nonpermanent yet very long term cell cycle arrest. Such on-off behavior of irradiated HLEC1 cells (i.e., to be stimulated to divide or contrarily to be inactivated) appears very unique. Though whether our present findings obtained with HLEC1 can be generalized in primary human diploid lens epithelial cells from different donors needs to be further clarified, our *in vitro* system would surely be invaluable to evaluate manifestations and mechanisms of such unique behavior of primary human diploid lens epithelial cells. A question of why cataractogenic potential of neutrons and heavy ions is very high remains fully open despite significant relevance to radiation protection, radiation therapy and space missions [3], and our *in vitro* system may help answer this fascinating question.

## Supporting Information

Figure S1 **Methods used to choose clonogenic colonies for the analysis and evaluate its area.**
(TIF)

Figure S2 **The frequency distribution of area of clonogenic colonies arising from HLEC1 and WI-38.**
(TIF)

Figure S3 **The fraction of clonogenic colonies exceeding the mean+2SD area of sham-irradiated controls among all plated cells.**
(TIF)

Figure S4 **Methods used to evaluate cell numbers in each clonogenic colony arising from HLEC1.**
(TIF)

Figure S5 **The integrated density of clonogenic colonies arising from HLEC1 and WI-38.**
(TIF)

Figure S6 **The relationship between the integrated density and cell numbers in all countable clonogenic colonies arising from HLEC1.**
(TIF)

Figure S7 **Alterations in the distribution of colonies in HLEC1 and AG01522D.**
(TIF)

Table S1 **Number of cells replated per 10-cm dish after irradiation for colony formation.**
(PDF)
