# Supplementary figures and images for: Ionizing Irradiation Not Only Inactivates Clonogenic Potential in Primary Normal Human Diploid Lens Epithelial Cells but Also Stimulates Cell Proliferation in a Subset of This Population
Source: PLoS One. 2014 May 19;9(5):e98154. doi: 10.1371/journal.pone.0098154 (PMC4026537; doi:10.1371/journal.pone.0098154)

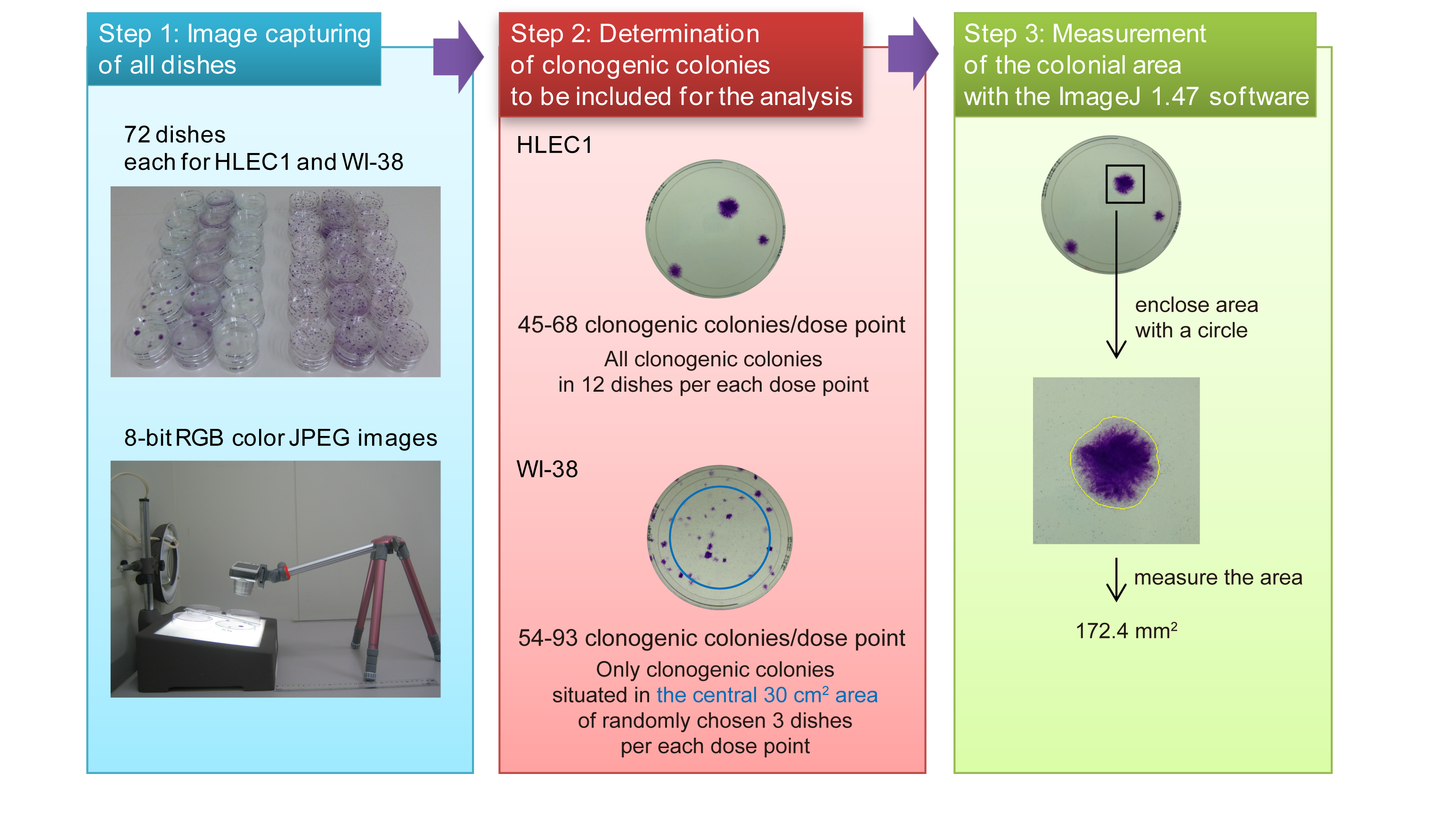

Supplement: Figure S1 — Methods used to choose clonogenic colonies for the analysis and evaluate its area. (TIF) [file pone.0098154.s001.tif]

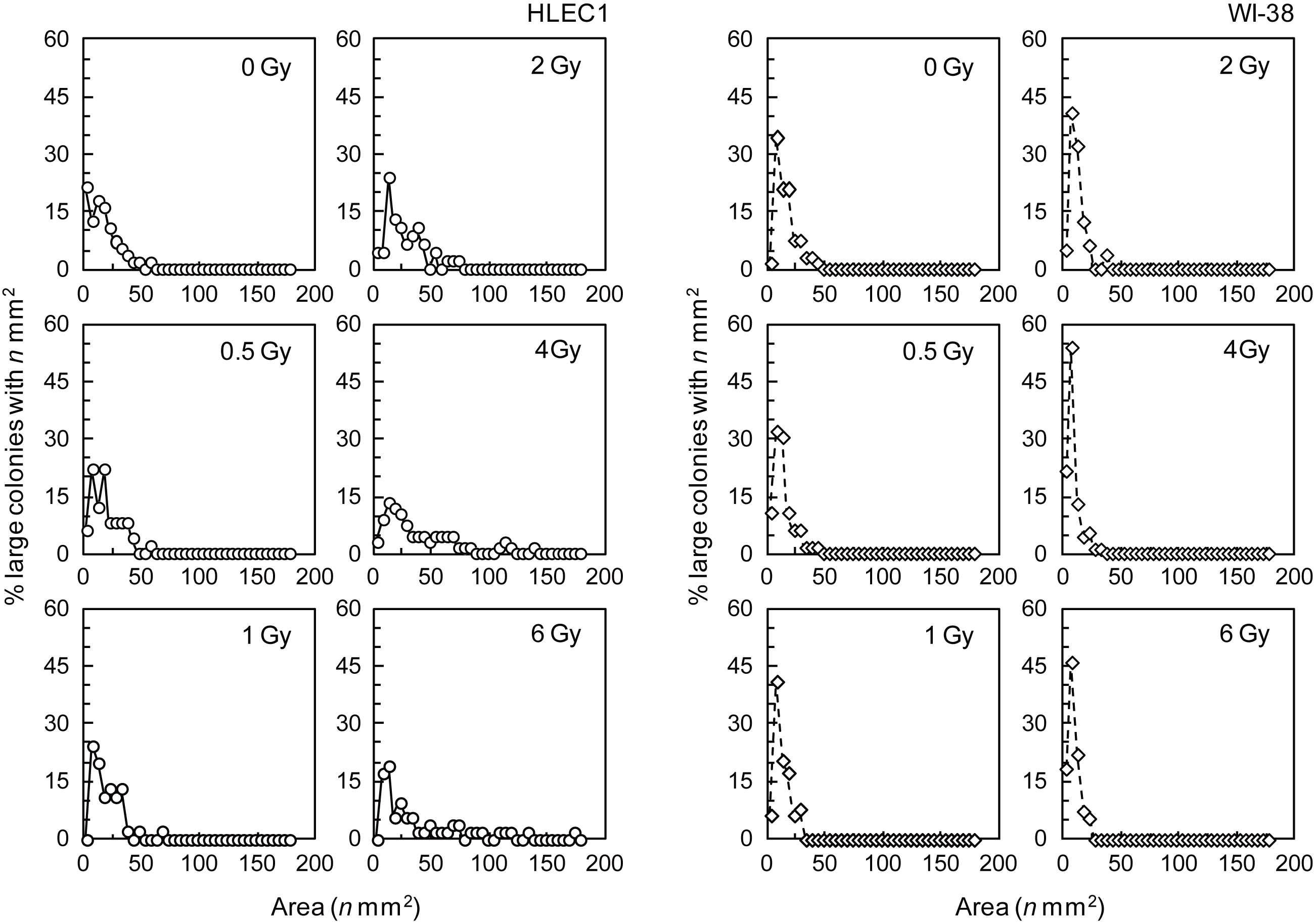

Supplement: Figure S2 — The frequency distribution of area of clonogenic colonies arising from HLEC1 and WI-38. (TIF) [file pone.0098154.s002.tif]

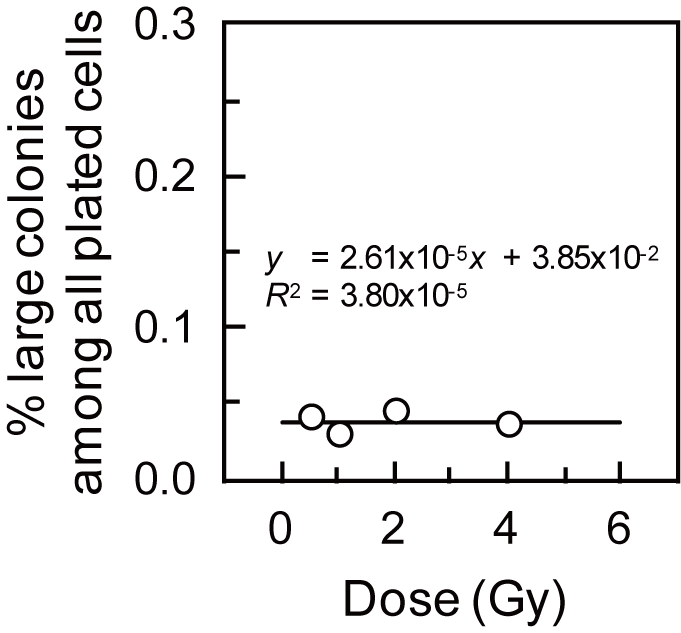

Supplement: Figure S3 — The fraction of clonogenic colonies exceeding the mean+2SD area of sham-irradiated controls among all plated cells. (TIF) [file pone.0098154.s003.tif]

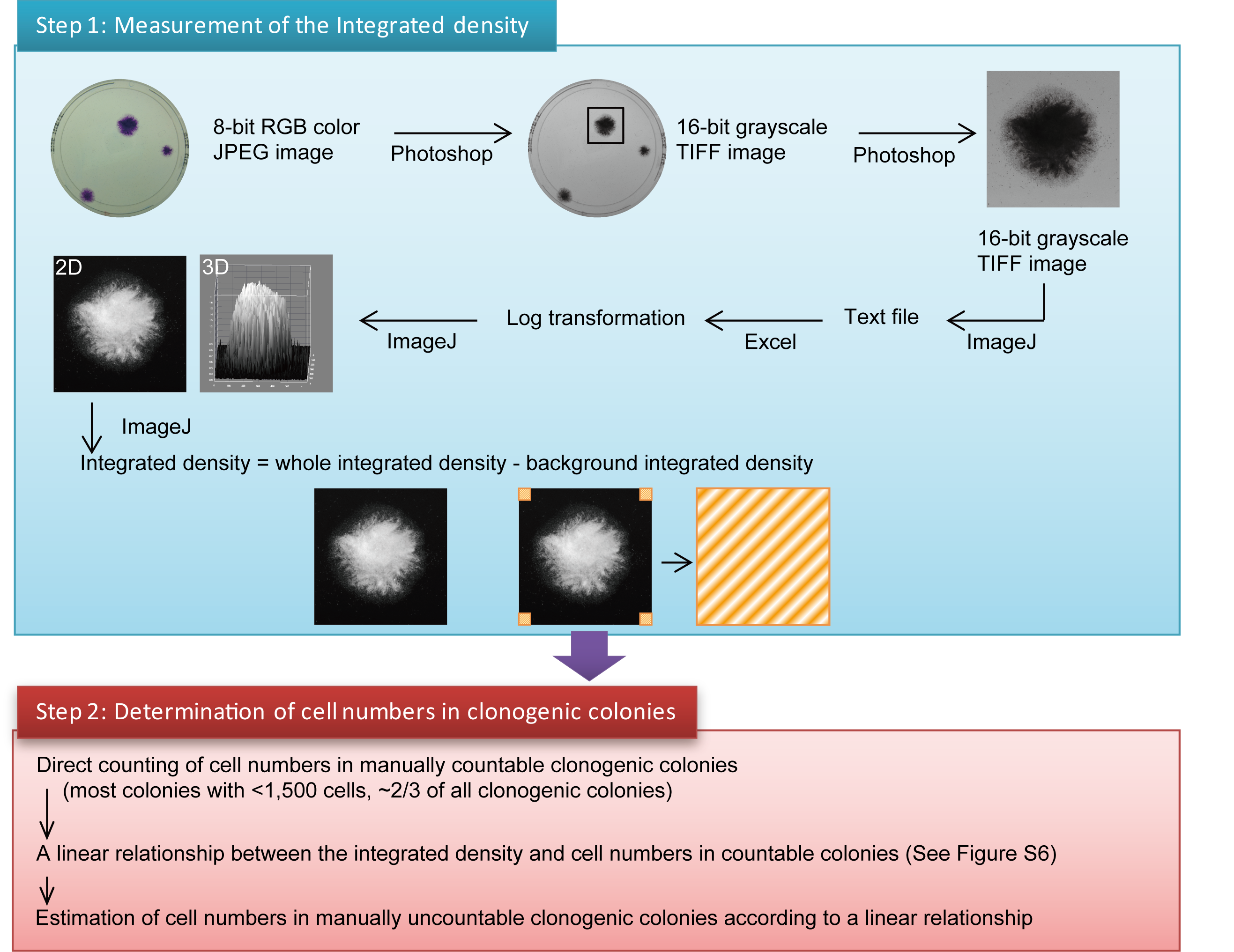

Supplement: Figure S4 — Methods used to evaluate cell numbers in each clonogenic colony arising from HLEC1. (TIF) [file pone.0098154.s004.tif]

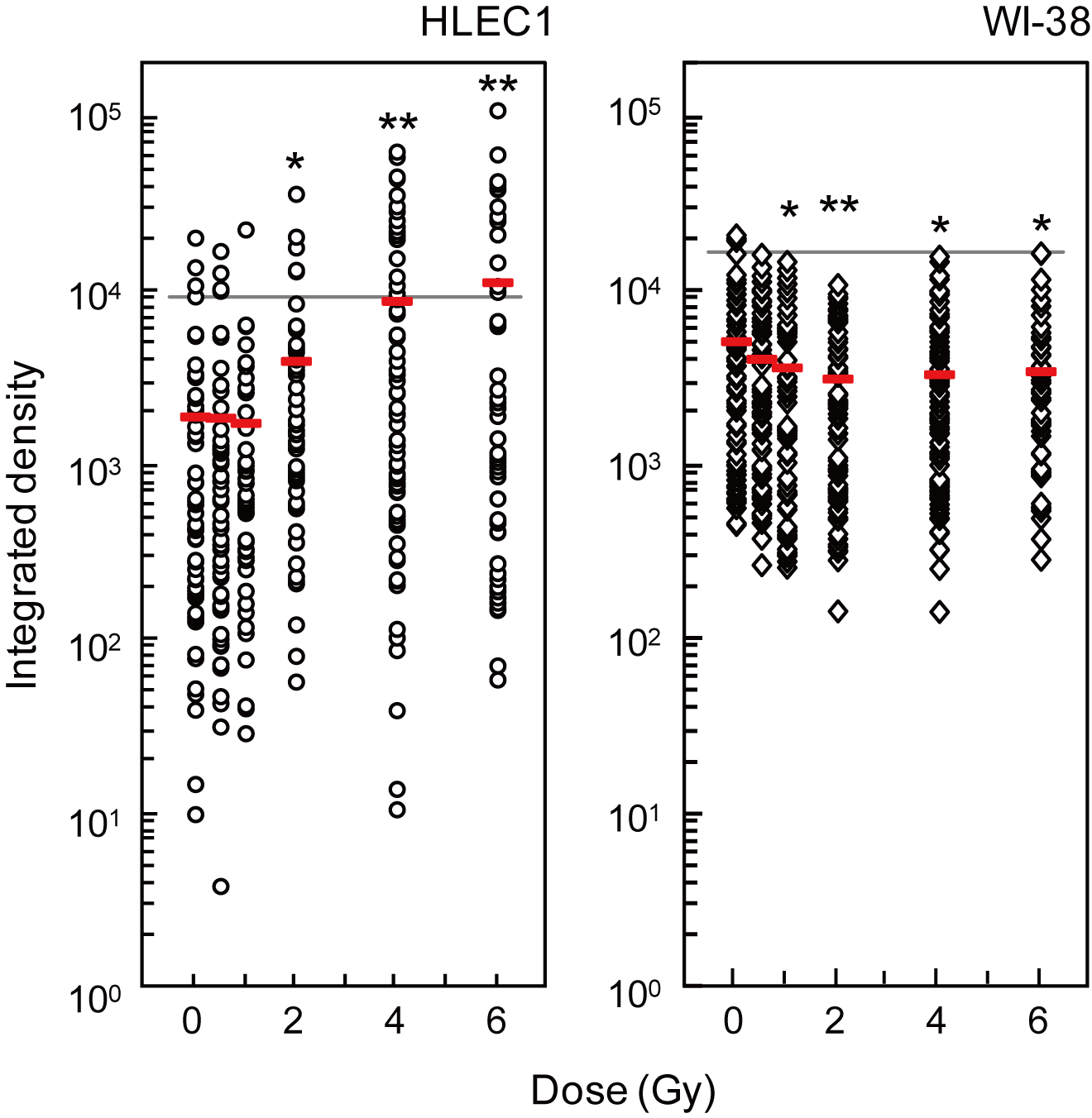

Supplement: Figure S5 — The integrated density of clonogenic colonies arising from HLEC1 and WI-38. (TIF) [file pone.0098154.s005.tif]

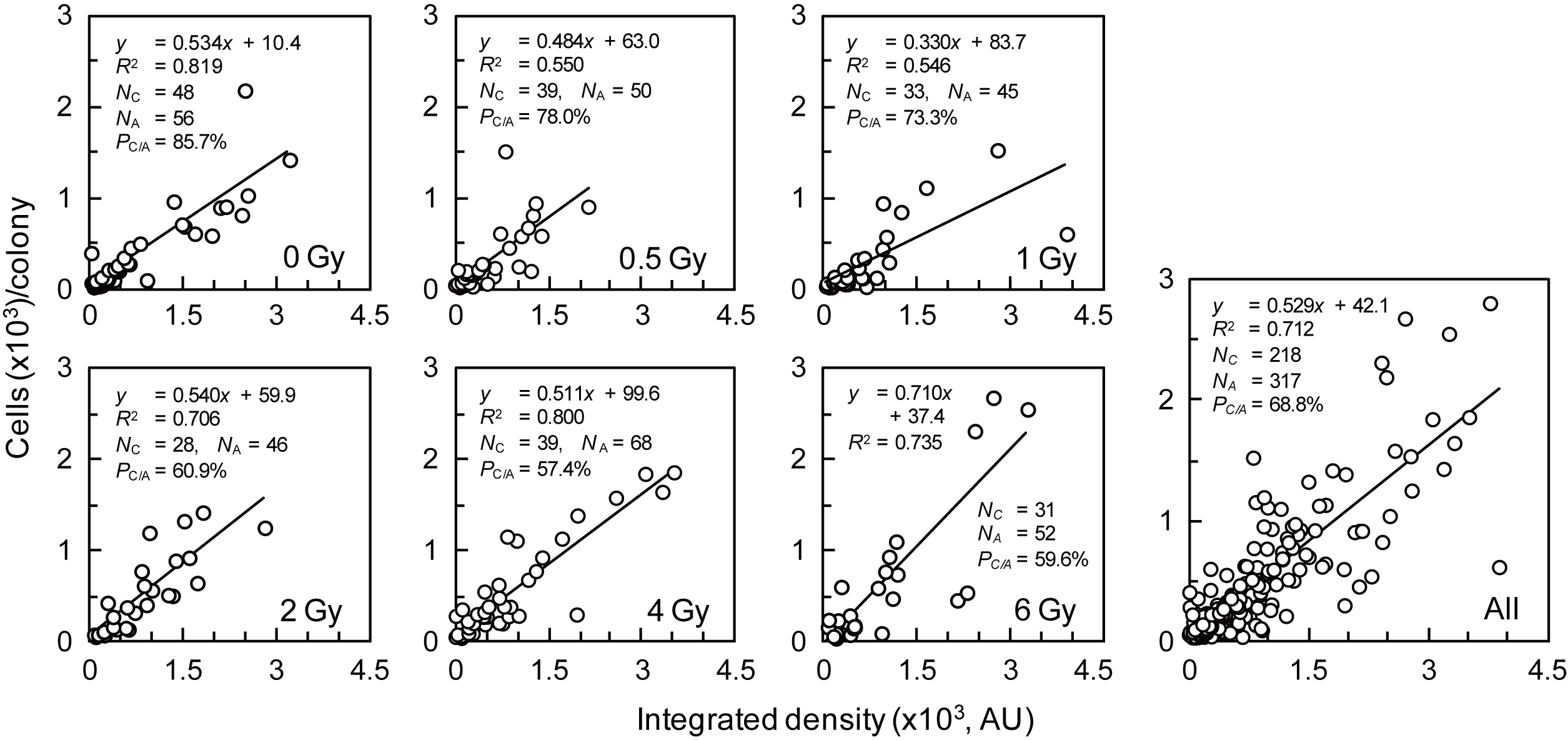

Supplement: Figure S6 — The relationship between the integrated density and cell numbers in all countable clonogenic colonies arising from HLEC1. (TIF) [file pone.0098154.s006.tif]

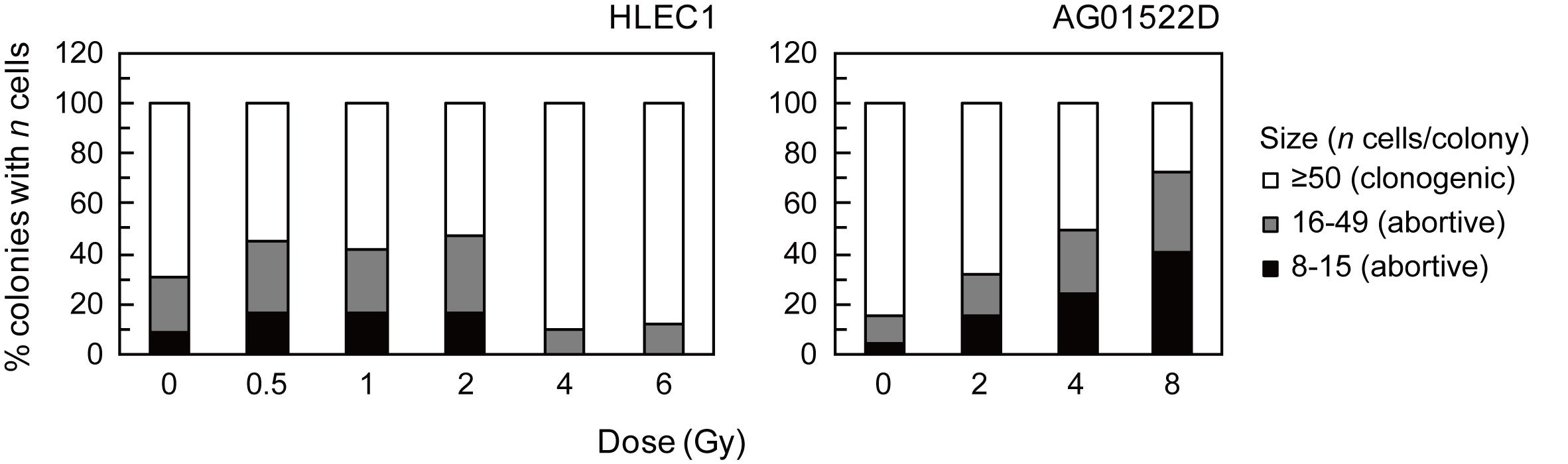

Supplement: Figure S7 — Alterations in the distribution of colonies in HLEC1 and AG01522D. (TIF) [file pone.0098154.s007.tif]
