# Supplementary material for: Ionizing Irradiation Not Only Inactivates Clonogenic Potential in Primary Normal Human Diploid Lens Epithelial Cells but Also Stimulates Cell Proliferation in a Subset of This Population
Source: PLoS One. 2014 May 19;9(5):e98154. doi: 10.1371/journal.pone.0098154 (PMC4026537; doi:10.1371/journal.pone.0098154)
Supplement: Table S1 — Number of cells replated per 10-cm dish after irradiation for colony formation. (PDF) [file pone.0098154.s008.pdf]

Table S1. Number of cells replated per 10-cm dish after irradiation for colony formation.

**Dose (Gy)**	**Cell types**		**Statistical significance**
	**HLEC1**	**WI-38**	
0	300 ± 2[a]	302 ± 3[a]	N.S.[b]
0.5	400 ± 4[a]	401 ± 4[a]	N.S.[b]
1	550 ± 2[a]	550 ± 1[a]	N.S.[b]
2	1099 ± 3[a]	1098 ± 3[a]	N.S.[b]
4	5206 ± 10[a]	5194 ± 9[a]	N.S.[b]
6	14956 ± 5202[a]	17775 ± 4100[a]	N.S.[b]

[a]The data represent means and SD of three independent experiments with quadruplicate measurements. [b]Non significant when the number of HLEC1 plated was compared with that of WI-38. *p* values ranged between 0.10 and 0.44.
